# Comparative Transcriptome Profiles of Human Blood in Response to the Toll-like Receptor 4 Ligands Lipopolysaccharide and Monophosphoryl Lipid A

**DOI:** 10.1038/srep40050

**Published:** 2017-01-05

**Authors:** Liming Luan, Naeem K. Patil, Yin Guo, Antonio Hernandez, Julia K. Bohannon, Benjamin A. Fensterheim, Jingbin Wang, Yaomin Xu, Perenlei Enkhbaatar, Ryan Stark, Edward R. Sherwood

**Affiliations:** 1 Department of Anesthesiology, Vanderbilt University Medical Center, Nashville, TN, USA; 2 Department of Pathology, Microbiology and Immunology, Vanderbilt University Medical Center, Nashville, TN, USA; 3 Department of Biostatistics, Vanderbilt University Medical Center, Nashville, TN, USA; 4 Department of Anesthesiology, the University of Texas Medical Branch, Galveston, TX, USA; 5 Department of Pediatrics, Vanderbilt University Medical Center, Nashville, TN, USA

## Abstract

Monophosphoryl lipid A (MPLA), a less toxic derivative of lipopolysaccharide (LPS), is employed as a vaccine adjuvant and is under investigation as a non-specific immunomodulator. However, the differential response of human leukocytes to MPLA and LPS has not been well characterized. The goal of this study was to compare the differential transcriptomic response of human blood to LPS and MPLA. Venous blood from human volunteers was stimulated with LPS, MPLA or vehicle. Gene expression was determined using microarray analysis. Among 21,103 probes profiled, 136 and 130 genes were differentially regulated by LPS or MPLA, respectively. Seventy four genes were up-regulated and 9 were down-regulated by both ligands. The remaining genes were differentially induced by either agent. Ingenuity Pathway Analysis predicted that LPS and MPLA share similar upstream regulators and have comparable effects on canonical pathways and cellular functions. However, some pro-inflammatory cytokine and inflammasome-associated transcripts were more strongly induced by LPS. In contrast, only the macrophage-regulating chemokine CCL7 was preferentially up-regulated by MPLA. In conclusion, LPS and MPLA induce similar transcriptional profiles. However, LPS more potently induces pro-inflammatory cytokine and inflammasome-linked transcripts. Thus, MPLA is a less potent activator of the pro-inflammatory response but retains effective immunomodulatory activity.

Lipopolysaccharide (LPS, endotoxin) is an integral component of the outer membrane of Gram negative bacteria and plays an essential role in membrane structural integrity^1^,^2^. LPS is comprised of a hydrophobic lipid A component that serves to anchor LPS in the membrane, a non-repeating core oligosaccharide and a distal polysaccharide, known as the O-antigen, that interacts with the extracellular environment and contributes to morphological traits of Gram negative bacteria^3^,^4^. Upon lysis of Gram negative bacteria, LPS can be released into the host environment where it activates the innate immune system by binding to the toll-like receptor 4 (TLR4) complex on macrophages, dendritic cells, neutrophils, endothelial cells and B lymphocytes^5^,^6^. Lipid A is the component of LPS that binds the TLR4 complex to initiate cellular activation^7^. As such, lipid A mediates stimulation of the innate immune response and induces a robust inflammatory response characterized by cytokine and chemokine secretion, increased expression of leukocyte adhesion molecules, leukocyte recruitment, alterations in vascular permeability and changes in vascular tone^8^. Thus, lipid A is the major component of Gram negative bacteria that is recognized by the immune system to facilitate the host response to infection. Systemic administration of LPS or lipid A induces a syndrome characterized by inflammation, hemodynamic instability, metabolic dysfunction and organ injury that is termed endotoxin shock and mimics many of the alterations present during severe sepsis and septic shock^9^,^10^.

In addition to initiating the host response to Gram negative infection, LPS possesses immunomodulatory properties and, upon prior exposure, can modify the host response to subsequent LPS exposure or infection. Treatment with LPS will induce a state known as endotoxin tolerance in which the production of pro-inflammatory mediators is greatly attenuated in response to a subsequent LPS challenge^11^,^12^. The induction of endotoxin tolerance has been shown to modulate the LPS-induced pro-inflammatory response and to endow protection from a normally lethal LPS challenge^13^. Other studies show that LPS priming will augment the host response to infection with *Staphylococcus aureus, Pseudomonas aeruginosa* and polymicrobial peritonitis induced by cecal ligation and puncture (CLP)^12^,^14^. The enhanced resistance to infection was associated with improved bacterial clearance and attenuated cytokine production. Thus, LPS possesses immunomodulatory properties that could be useful in augmenting the host response to infection in high risk and vulnerable patient populations.

Unfortunately, LPS is not an attractive agent for clinical use, owing to its toxicity in humans. However, the lipid A analog monophosphoryl lipid A (MPLA) provides a useful alternative. MPLA was initially prepared by hydrolysis of lipid A from *Salmonella enterica* serotype Minnesota Re 595 resulting in removal of the *O*-1 phosphate group. Removal of the *O*-1 phosphate group results in a product with low pro-inflammatory activity but potent immunomodulatory effects^15^. As such, MPLA is currently employed as a component of FDA-approved vaccine adjuvant systems and is used in commercially available vaccines against papilloma viruses^16^. MPLA also has potent effects on the innate immune system. MPLA elicits endotoxin tolerance, and attenuates the pro-inflammatory cytokine surge and hemodynamic alterations in response to subsequent LPS challenge^17^,^18^. Studies from our research group have consistently shown that priming with MPLA augments the innate host response to infection in murine models of sepsis, including CLP-induced bacterial peritonitis and *Pseudomonas* burn wound infection, leading to improved survival outcomes^19^,^20^,^21^.

Although LPS and MPLA have different impacts on innate immune system activation, the gene expression profiles induced by LPS and MPLA in humans have not been compared. We hypothesized that MPLA, when compared to LPS, is less potent in inducing expression of pro-inflammatory cytokine genes while maintaining potent adjuvant properties and the ability to augment innate antimicrobial immunity. The main objective of the current study was to examine the differential transcriptomic response of human peripheral blood to LPS and MPLA using microarray and Ingenuity Pathway Analysis (IPA) to better understand differences in the biological responses to each agent. This study enabled us to identify functional attributes of the differentially expressed transcripts and to uncover the interactions among the differentially expressed genes within treatment groups and with other molecules in the IPA database. To our knowledge, this is the first study to compare the transcriptomic profiles in human blood challenged with LPS and MPLA. Our findings have significant translational impact, because MPLA is an attractive immunomodulatory agent with application as a vaccine adjuvant and non-specific modifier of innate immune responses to infection.

## Results

### Microarray

The data discussed in this publication have been deposited in NCBI’s Gene Expression Omnibus and are accessible through GEO Series accession number GSE72557.

### Differentially Expressed Genes after LPS or MPLA Treatment

**Figure 1:**
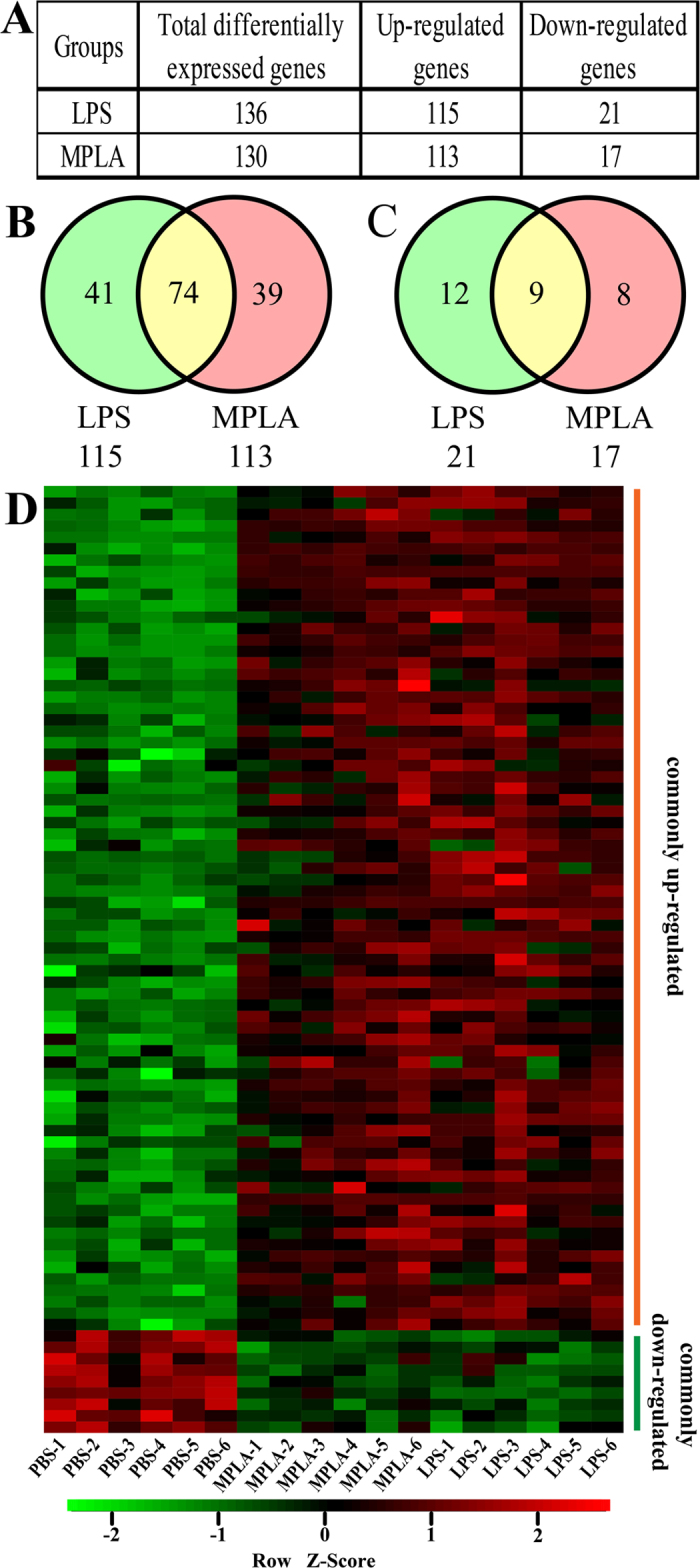
Lipopolysaccharide and Monophosphoryl Lipid A Induce Similar Transcriptome Profiles in Human Blood. (**A**) Numbers of differentially expressed genes after LPS or MPLA treatment. (**B**) Venn diagram indicating the overlap of genes that were significantly upregulated after LPS and MPLA treatment. (**C**) Venn diagram indicating the overlap of genes that were significantly downregulated after LPS and MPLA treatment. (**D**) Heat map of the hierarchical clustering of commonly regulated genes depicting their expression patterns and variation in human blood samples treated with LPS, MPLA, or vehicle control (PBS). The color key indicates the direction of changes, with red depicting genes significantly up-regulated and green showing genes significantly down-regulated. Genes were clustered based on their expression values across samples using Pearson correlation and complete linkage function.

Gene expression profiles in LPS- or MPLA-treated human blood samples were assessed using microarray analysis. Among 21,103 qualified probes present in the microarray, compared to the control PBS group, 136 and 130 genes were differentially expressed (fold change > 2) in human blood when stimulated by LPS or MPLA, respectively. Out of 136 differentially expressed genes in LPS group, 115 genes were significantly up-regulated and 21 were significantly down-regulated. In the MPLA group, 113 of 130 differentially expressed genes were up-regulated and 17 down-regulated (Fig. 1A). Of note, in both groups, the number of up-regulated genes was approximately 6 times higher than that of down-regulated genes. Meanwhile, the numbers of total differentially expressed, up-regulated, and down-regulated genes were comparable between these two treatments.

To further assess the similar effects of these two TLR4 ligands, Venn diagrams were used to depict the overlap of genes that were significantly up- and down-regulated in human blood after LPS or MPLA treatment. Among up-regulated genes, 74 genes were commonly regulated by both TLR4 ligands, 41 genes were preferentially induced by LPS and 39 genes by MPLA (Fig. 1B). Among the down-regulated genes, expression of 9 genes was regulated by both LPS and MPLA, while 12 genes were preferentially induced by LPS and 8 by MPLA (Fig. 1C). Furthermore, hierarchical clustering of these 83 commonly up- and down-regulated genes in blood samples treated with PBS, LPS and MPLA resulted in three distinct clusters distinguishing the six MPLA-treated and six LPS-treated blood samples from the remaining six control samples (Fig. 1D).

A list of commonly and differentially induced genes by LPS and MPLA is provided in Supplemental Tables S1, S2 and S3. Total up- or down-regulated genes by LPS or MPLA are shown in Supplemental Tables S4, S5, S6 and S7.

### Validation of Gene Array Data by qPCR

**Figure 2:**
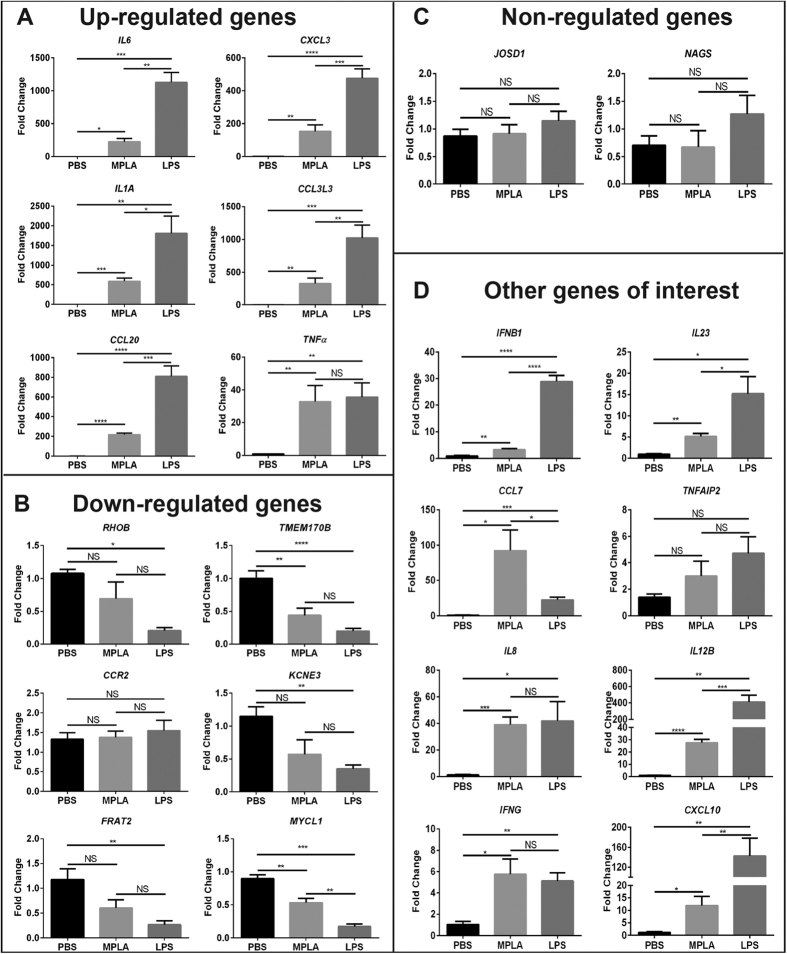
Validation of Microarray Data by Quantitative Real-Time PCR. (**A**) qPCR validation of six genes randomly chosen from top up-regulated genes in our genomic analysis. (**B**) qPCR validation of six genes randomly chosen from most down-regulated genes in our genomic analysis. (**C**) qPCR validation of two random non-regulated genes. (**D**) qPCR validation of eight genes of interest chosen based on their expression patterns. ΔΔCt values graphed are relative to the endogenous controls *HPRT1* with SEM. *p < 0.05, **p < 0.01, ***p < 0.001, ****p < 0.0001, NS = not significant; n = 4 in each group, and qPCR was performed in triplicate.

To verify the data from microarray analysis, the expression of six of the top up-regulated genes (*IL6, CCL20, CXCL3, IL1A, TNFα,* and *CCL3L3*), six of top down-regulated genes (*RHOB, TMEM170B, CCR2, KCNE3, FRAT2*, and *MYCL1*), two random non-regulated genes (*JOSD1* and *NAGS*), and eight genes of interest (*IFNB1, IL23, CCL7, TNFAIP2, IL8, IL12B, IFNG*, and *CXCL10*), based on their role in the inflammatory response and differential expression after LPS or MPLA challenge, were examined by quantitative Real-Time PCR (qPCR). The relative expression levels of tested genes exhibited the same regulatory trends as compared with microarray analysis, with exception of *CCR2* and *TNFAIP2* (Fig. 2). Among each of the up-regulated and down-regulated genes, LPS induced a significantly greater effect as compared to MPLA (Fig. 2A and B). There was no significant difference among groups in the expression of two randomly chosen “non-regulated” genes (JOSD1 and NAGS) (Fig. 2C). Among the genes of interest to us, the regulatory trends were true between qPCR and microarray assays for *IFNB1, IL23, CCL7, IL8, IL12B, IFNG* and *CXCL10*, but not for *TNFAIP2* (Fig. 2D). There was no difference in the levels of *CCR2* and *TNFAIP2* genes among all the groups (Fig. 2B and D).

### Canonical Pathways Modulated by LPS and MPLA

**Figure 3:**
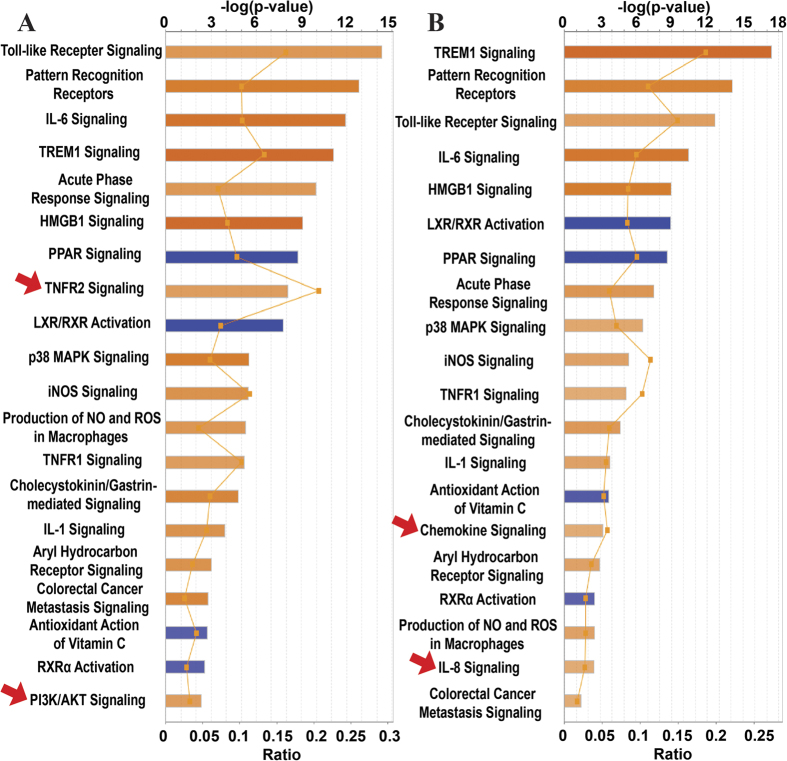
Canonical Pathways Modulated by Differentially Expressed Genes after LPS or MPLA Treatment. Ingenuity pathway analysis showing significantly altered canonical pathways modulated by the 136 and 130 differentially expressed genes after LPS (**A**) and MPLA (**B**) treatment, respectively. The pathways are indicated on the y-axis. On the x-axis, the significance score (negative log of P-value calculated using Fisher exact test) for each pathway is indicated by the bars, and the line represents the ratio of genes in a given pathway that meet the cut-off criteria among total genes that make up that pathway. The bars in the chart are colored to indicate their activation z-scores. Orange bars predict an overall increase in the activity of the pathway while blue bars indicate a prediction of an overall decrease in activity. The entries that have a −log (p-value) greater than 1.3 and an absolute z-score value greater than 2 are displayed. Arrows, the pathways that are activated by LPS or MPLA only.

The 136 and 130 genes whose mRNA levels in human blood were affected by LPS or MPLA, respectively, were submitted to IPA software analysis, and specific changes in biological pathways detected by a core analysis are shown in Fig. 3. Our pathway-based analysis revealed that both LPS and MPLA activated 14 pathways, such as Toll-like receptor signaling, role of pattern recognition receptors, IL-6 signaling, TREM1 signaling, and acute phase response signaling. Besides these shared pathways, TNFR2 signaling and PI3K/AKT signaling pathway genes were preferentially induced by LPS (Fig. 3A, arrows), while genes associated with chemokine signaling and IL-8 signaling were selectively induced by MPLA (Fig. 3B, arrows). On the other hand, 4 pathways (LXR/RXR activation, PPAR signaling, antioxidant action of vitamin C, and PPARα/RXRα activation) were commonly inhibited by LPS (Fig. 3A) and MPLA (Fig. 3B).

### Networks

**Figure 4:**
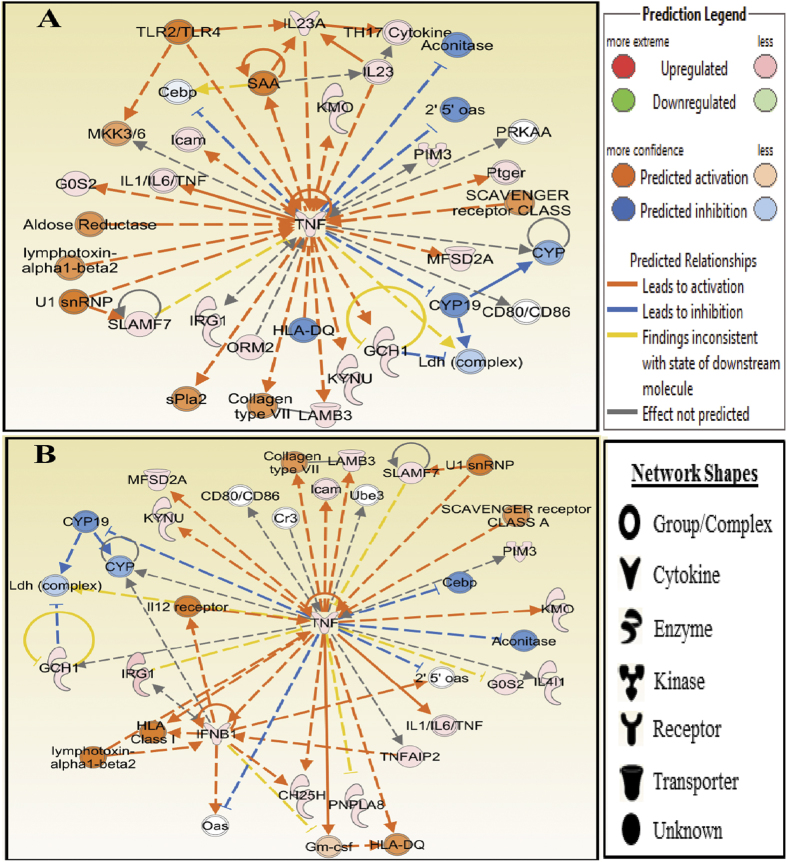
Tumor Necrosis Factor-alpha (TNF-α) Networks Identified by Ingenuity Analysis in LPS and MPLA Treated Human Blood. The molecular network of TNF-α identified via Ingenuity analysis in LPS (**A**) and MPLA (**B**) treated groups. The analysis was performed *in silico* using Molecular Activity Predictor analysis of IPA. The genes are represented as colored nodes. The red nodes represent the upregulated genes, while the blue nodes represent the downregulated genes. Color intensity reflects magnitude of change. Genes without color were not affected by the treatment. The network diagram shows the biological relationship between the indicated genes lines: — represents direct physical interactions; ----- represents indirect functional interactions; → represents activation; ┤represents inhibition. The blue lines indicate that the direction of regulation is consistent with IPA prediction. In contrast, yellow lines indicate that the regulation observed is inconsistent with expectations, while grey lines indicate lack of pre-existing data to formulate expectations. Nodes are displayed using various shapes that represent the functional class of the genes.

We further identified the networks *in silico* using Molecular Activity Predictor analysis of IPA with the differentially expressed genes in human blood treated with LPS or MPLA. In both groups, the top predicted network is centered on tumor necrosis factor-alpha (TNF-α), and the network diagrams are shown in Fig. 4. TNF-α is one of the most important and earliest induced cytokines in an inflammatory insult, which activates the TNF-α receptor involved in innate immune cell function. Our data revealed that the TNF-α network in LPS-treated human blood consists of 12 genes (Fig. 4A), which are predicted to be mainly associated with Small Molecule Biochemistry (*G0S2, GCH1, KMO, KYNU*, and *TNF*), Amino Acid Metabolism (*GCH1, KMO, KYNU*, and *TNF*), and Cardiovascular System Development and Function (*GCH1, IL23A, PIM3*, and *TNF*). Whereas, the TNF-α network in MPLA-treated human blood consists of 15 genes (Fig. 4B), which are predicted to be mainly associated with Cell-To-Cell Signaling and Interaction (*CH25H, CYP, IFNB1*, and *TNF*), Cellular Growth and Proliferation (*Gm-csf, IFNB1, IL4I1, PIM3, SLAMF7*, and *TNF*), and Hematological System Development and Function (*CH25H, Gm-csf, IFNB1, IL4I1, PIM3, SLAMF7*, and *TNF*). Similar patterns were found in the two network diagrams. For example, there were five genes predicted to be inhibited by TNF in both groups, and four of which are common among them (*2*′*5*′*oas, Aconitase, CEBP*, and *CYP19*). In addition, the same interactions between *TNF* and *CYP, CYP19*, Ldh (complex), and *GCH1* were observed in both treatments.

### Functional Analysis

**Figure 5:**
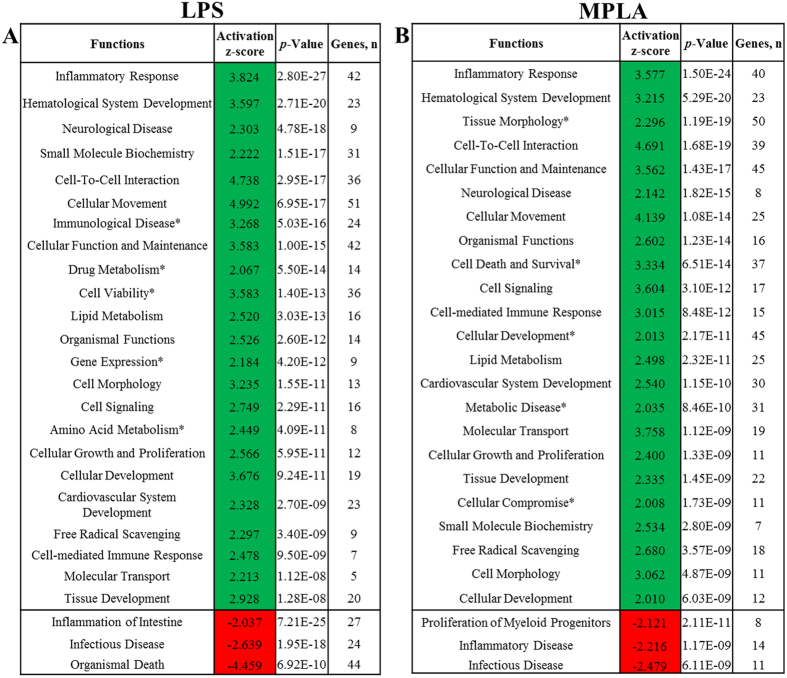
Functional Analysis. Differentially expressed genes in LPS (**A**) and MPLA (**B**) treated human blood were used for Functional analysis in IPA software. Only functional annotations that obtained a Regulation z-score value higher than the absolute value of 2, which is considered significant and therefore for which IPA could predict the Activation State, are presented. Green indicates functions that were up-regulated and red indicates functions that were down-regulated. Asterisks, the functions that are activated by LPS or MPLA only. P-value < 0.05 calculated by Fisher’s Exact test; “Genes, n” = number of genes associated to annotation in our datasets.

Using the datasets of modulated genes from the LPS and MPLA treatments, IPA Functional analysis generated predictions for significantly increased (z-score > 2) or decreased (z-score < −2) activity in various cellular processes. The major altered biological functions are summarized in Fig. 5. In LPS treated human blood, activity of 23 biological functions was predicted to be increased. Those LPS-modulated genes were mostly related to cellular movement (51 genes), inflammatory response (42 genes), and cellular function and maintenance (42 genes). Whereas, inflammation of intestine (27 genes), infectious disease (24 genes), and organismal death (24 genes) were the only three decreased functions predicted by IPA in LPS-treated human blood (Fig. 5A). Whereas there were 23 biological functions predicted to be increased upon MPLA treatment in human blood. Among them were functions related to tissue morphology (50 genes), cellular function and maintenance (45 genes), and cellular development (45 genes). The three decreased functions predicted by IPA in MPLA treated human blood were related to proliferation of myeloid progenitor cells (8 genes), inflammatory disease (14 genes), and infectious disease (11 genes) (Fig. 5B).

### Upstream Analysis

**Figure 6:**
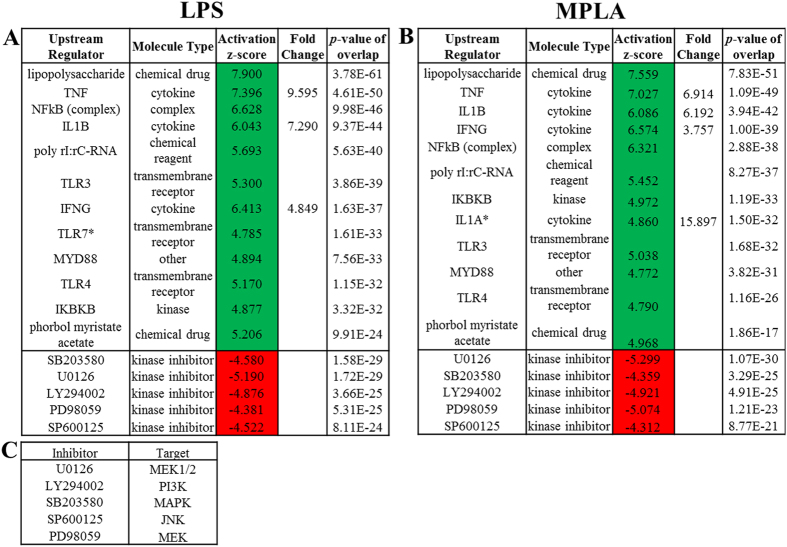
Upstream Analysis. The top 12 activated and top 5 inhibited upstream regulators in LPS- (**A**) or MPLA-treated (**B**) human blood predicated by Upstream analysis in IPA software are shown in the table. The prediction of activation state is based on the global direction of changes of the modulated genes. The activation Z-score, which indicates whether the observed gene responses to upstream regulators agree with expected changes derived from the literature that accrued in the Ingenuity® Knowledge Base, was used to predict the activation state. Z-scores ≥ 2 or ≤ −2 indicates that the upstream regulator was predicted to be activated or inhibited, respectively. A Fisher’s Exact Test was used to determine the significance of the overlap between the regulator and the LPS- and MPLA-responsive genes. The targets of the inhibitors are listed (**C**). Asterisks, the upstream regulators that are activated by LPS or MPLA only.

The Upstream analysis section of the core analysis was used to identify the activation state of upstream regulators that could explain the observed gene expression profile alterations in LPS- and MPLA- treated human blood. The predicted top 12 activated and top 5 down-regulated regulators in human blood treated with TLR4 ligands are shown in Fig. 6. High similarities were observed between LPS and MPLA groups as evidenced by the fact that there is only one distinct upstream regulator presented in each group, i.e., TLR7 was predicted to be activated only in LPS- (Fig. 6A, *) while IL1A only in MPLA-treated (Fig. 6B, *) human blood. Lipopolysaccharide (LPS) and TNF were predicted the most relevant activated upstream regulators in both treatments. Genes and molecules that are known to induce the activation of inflammatory genes were found among the predicted activated upstream regulators, and they were also found up-regulated in our data sets. For example, as compared to the PBS control group, *TNF, IL1B*, and *IFNG* were upregulated by 9.595, 7.290, and 4.849 folds, respectively, in LPS-treated human blood (Fig. 6A), and 6.914, 6.192, and 3.757 folds, respectively, in MPLA-treated samples (Fig. 6B). Those predicted activated regulators included several chemical drugs, cytokines, and transmembrane receptors. The top predicted inhibited regulators included five kinase inhibitors (SB203580, U0126, LY294002, PD98059, and SP600125), and their targets are listed in Fig. 6C.

### Genes that are Preferentially Modulated by LPS or MPLA

**Figure 7:**
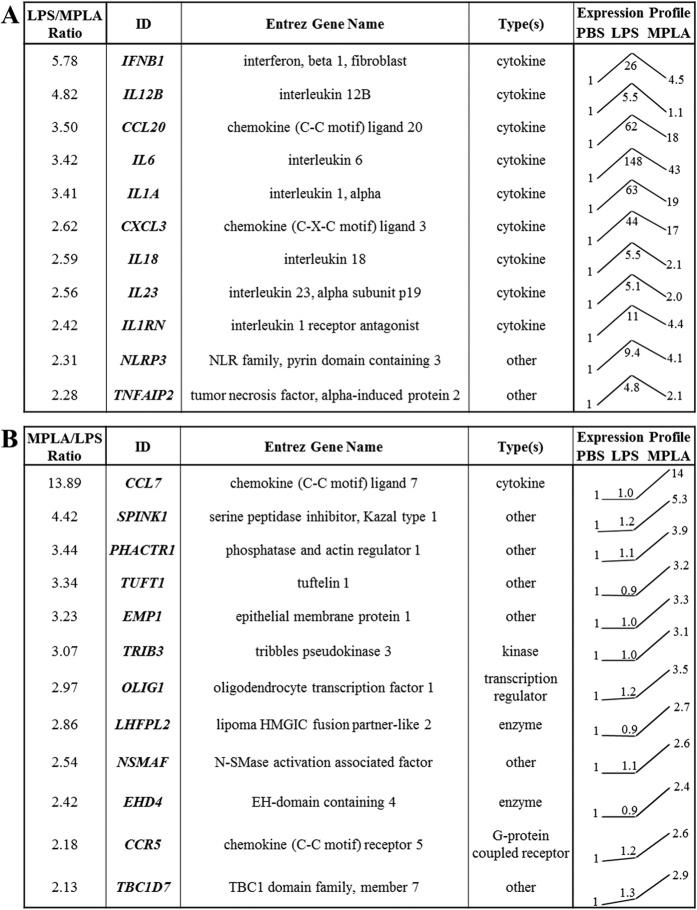
Comparison of Genes Induced by LPS and MPLA. Eleven more strongly induced genes by LPS (**A**) and twelve more preferentially modulated genes by MPLA (**B**) are shown in the tables. Normalized intensity value of each gene from microarray hybridization profiles were further normalized to that of the corresponding PBS sample, and then the relative expression trend was shown as a line among the 3 groups (PBS, LPS, and MPLA). The relative expression ratio for each gene between the two treatments was further calculated, and only the genes with a ratio of 2 or over were displayed.

Despite the vast similar genomic response of human peripheral blood to LPS and MPLA, a further comparison of the effects of MPLA and LPS on human blood was performed. The genes that were more strongly induced by LPS or MPLA are listed in Fig. 7. Among the 11 genes that were more responsive to LPS, 10 of them were cytokines or chemokines, including a number of inflammatory mediators (*IFNB1, IL12B, IL1A, IL18*, and *IL23*), and 2 were associated with the NLRP3 inlfammasome (Fig. 7A). In contrast, only one out of 12 genes that were preferentially modulated by MPLA is a cytokine, namely *CCL7*, a chemokine known to attract monocytes and regulate macrophage function, was not changed upon LPS challenge but was strongly upregulated by MPLA (*14-fold*) (Fig. 7B). Of note, the expression profile trends showed that, all of the LPS-more-responsive genes was also up-regulated by MPLA, but to a lower magnitude (Fig. 7A); whereas the MPLA-more-responsive genes were not significantly modulated by LPS (<1.3 fold) (Fig. 7B). In addition, *IL6* showed the greatest upregulation in both groups (Fig. 7A, and Supplemental Data S4 and S6).

**Figure 8:**
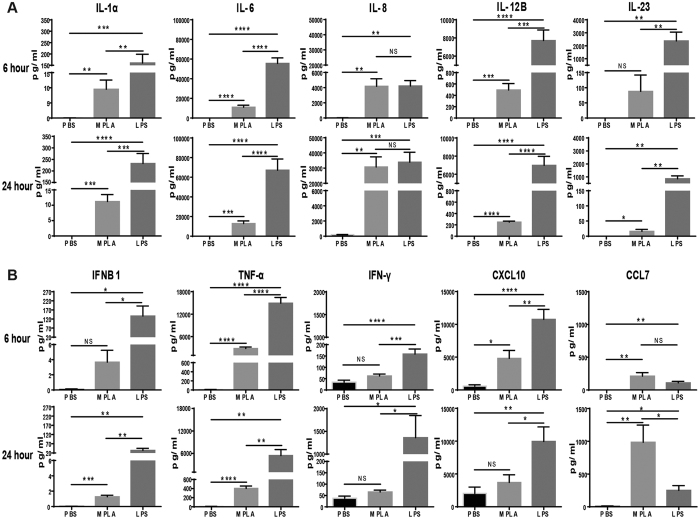
Cytokine Production by Human Peripheral Blood Mononuclear Cells (PBMC) After LPS or MPLA Treatments. PBMC were incubated in media supplemented with PBS, LPS or MPLA for 6 or 24 hours at 37 °C. Several interleukins (**A**) and cytokines (**B**) of interest were measured by ELISA and Bio-Plex analysis. *p < 0.05, **p < 0.01, ***p < 0.001, ****p < 0.0001, NS = not significant; n = 4 in each group, and data are representatives of three independent experiments.

Further studies were undertaken to validate the observed differences in gene expression after LPS and MPLA challenge at the protein level. Human peripheral blood mononuclear cells were incubated with LPS or MPLA for 6 or 24 hours at 37 °C and cytokine concentrations in conditioned media were measured. Concentrations of all measured cytokines, with the exception of CCL7 and IL-8, were more strongly induced by LPS than MPLA (Fig. 8). IL-8 was strongly and equally induced by both agents (Fig. 8A) and CCL7 production was more potently induced by MPLA (Fig. 8B). These results correlate with the qPCR data (Fig. 2).

**Figure 9:**
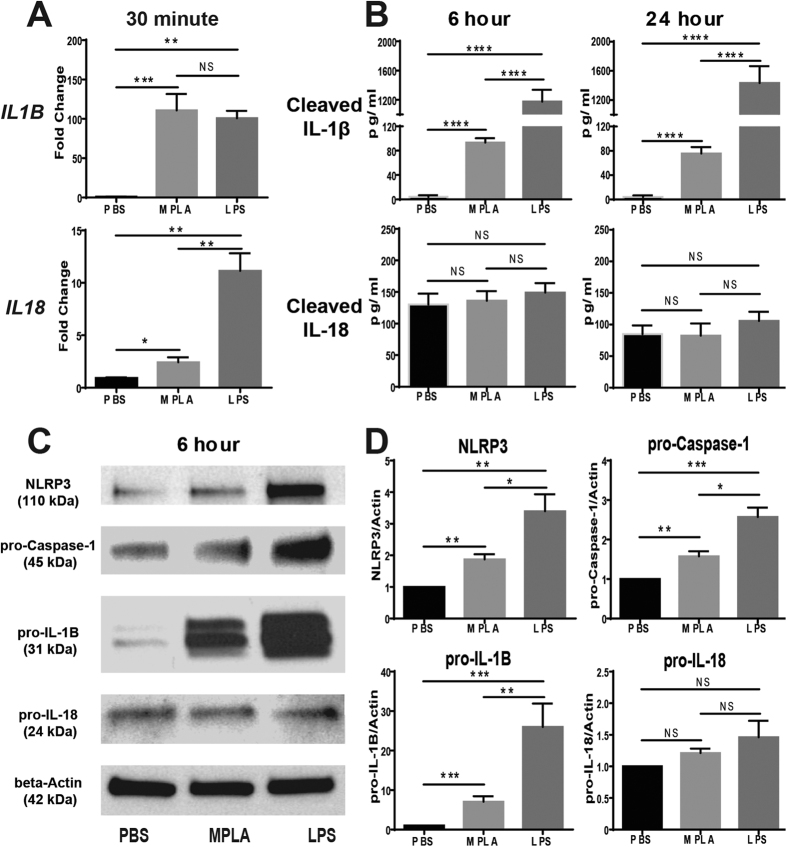
Expression of Inflammasome Components in Human PBMCs Treated with LPS or MPLA. (**A**) Gene expression of *IL1B* and *IL18* in human PBMCs 30 minutes after LPS or MPLA treatment. (**B**) Levels of cleaved IL-1β and IL-18 produced by human PBMCs 6 and 24 hours after LPS or MPLA treatment. (**C**) Expression of NLRP3 (110 kDa) and precursors of Caspase-1 (45 kDa), IL-1B (31 kDa), and IL-18 (24 kDa) in human PBMCs 6 hours after LPS or MPLA treatments. Gels have been cropped for clarity; the bands were confirmed by the comparison with full-length gel images and molecular weight (Supplementary Figure S8). (**D**) Densitometry analysis of the Western Blot results. *p < 0.05, **p < 0.01, ***p < 0.001, ****p < 0.0001, NS = not significant; n = 4 in each group, and data are representatives of three independent experiments.

The differential effects of LPS and MPLA on inflammasome activation was also further assessed (Fig. 9). To assess IL-1β and IL-18 transcription, peripheral blood mononuclear cells were incubated with LPS or MPLA for 30 minutes and gene expression was assessed by qPCR (Fig. 9A). IL-1β was equally induced by both agents whereas IL-18 expression was more potently induced by LPS. However, mature IL-1β concentrations were higher in conditioned media from peripheral blood mononuclear cells incubated with LPS than with mature MPLA whereas no difference in IL-18 concentration was observed between groups (Fig. 9B). Inflammasome-associated protein expression was further assessed by Western blotting after incubation of peripheral blood mononuclear cells with LPS or MPLA for 6 hours. Intracellular NLRP3, pro-Caspase-1 and pro-IL-1β were higher after incubation with LPS than with MPLA whereas as pro-IL-18 was not significantly different (Fig. 9C and D).

## Discussion

The major findings of this study are that LPS and MPLA induce very similar transcriptional profiles and the canonical pathways activated by both agents are comparable. However, among commonly induced gene products there were differences in magnitude of expression in some cases. In addition, although there was significant overlap in gene expression, each agent induced expression of gene products that were not induced by the other based on our cut-off criteria. The most prominent differences were in the increased level of pro-inflammatory gene expression induced by LPS, when compared to MPLA. LPS more potently (>2 fold over MPLA) induced expression of several pro-inflammatory cytokine transcripts including *IFNB1, IL12B, IL23, IL6, IL1A* and *IL18*. We also observed a 1.5-fold increase in *IL1B* transcript expression after LPS challenge compared to MPLA (data not shown) and LPS more potently induced expression of NLRP3 mRNA, which further prompted our investigation of inflammasome activation. Our results show that LPS more potently induces intracellular expression of NLRP3, pro-Caspase-1 and pro-IL-1β protein as well as secretion of IL-1β by isolated peripheral blood mononuclear cells compared to MPLA. Those observations suggest that the enhanced pro-inflammatory activity of LPS is due, in part, to its ability to more potently activate the NLRP3 inflammasome and initiate cleavage of pro-IL-1β. Interestingly, IL-18 expression was more potently induced by LPS at the mRNA level but no differences in IL-18 protein expression were observed between groups. Most types of native LPS are potent inducers of NLRP3 inflammasome activation and induce a potent pro-inflammatory response. In agreement with our results, Schülke and colleagues showed that, as compared to its parent molecule LPS, MPLA induced a qualitatively similar but significantly less potent pro-inflammatory immune response in *in vitro, ex vivo*, and *in vivo* human and mouse test systems^22^. They further successfully generated a novel fusion protein consisting of MPLA and a model allergen Ovalbumin (MPLA : Ova), and demonstrated that MPLA : Ova can boost Th1, Th2, and Th17 TC-derived cytokine secretion and hence induce both stronger innate and adaptive immune responses compared to the mixture of both components^23^. Based on experimental evidence, Chilton and colleagues have postulated that LPS and native diphosphoryl lipid A potently induce NLRP3 expression resulting in increased NLRP3 protein production and effective inflammasome assembly whereas weak NLRP3 induction, as seen after MPLA exposure, fails to elicit adequate NLRP3 protein production^24^,^25^. The functional importance of LPS-induced inflammasome activation *in vivo* was demonstrated by Vanden Berghe and colleagues^26^ who reported that both IL-1β/IL-18 and Caspase-1 knockout mice are highly resistant to LPS-induced shock. Our results support the contention that LPS is a potent stimulus for inflammasome activation whereas MPLA is not and extend previous studies by demonstrating these differences in human blood. All previous studies on the differential induction of inflammasome activation by LPS and MPLA were performed in mice.

Despite its limited ability to induce a pro-inflammatory response, MPLA retains immunomodulatory properties associated with LPS. Work from our laboratory, and others, shows that priming of mice with either LPS or MPLA will non-specifically augment host resistance to bacterial infections caused by Gram negative and Gram positive bacteria as well as polymicrobial sepsis induced by cecal ligation and puncture^12^,^19^,^20^,^27^. Both agents potently augment bacterial clearance mechanisms and potentiate neutrophil recruitment to sites of infection. LPS and MPLA also induce endotoxin tolerance and activate both the MyD88- and TRIF-dependent signaling pathways^21^,^28^,^29^,^30^. The immunomodulatory properties of MPLA have been harnessed for clinical use as a component of FDA-approved, commercially available vaccine adjuvant systems^31^,^32^. The present study supports the concept that LPS and MPLA activate many common canonical pathways and activate those pathways with similar intensity. Canonical pathways are distinct from networks in that they are generated prior to data input, are based on the literature, and do not change upon data input, whereas networks are generated *de novo* on the basis of the researcher’s own input data. Biological understanding of the function of genes in pathways, and the currently available lists of “canonical” pathways are evolving rapidly. Among the canonical pathways that were similarly induced by both agents were TLRs, TREM-1, pattern recognition receptors, IL-6, HMGB-1, the acute phase response and MAP kinase activation. Downregulated pathways included peroxisome proliferator-activated receptors (PPAR) and liver X/retinoid X receptors (LXR/RXR).

The similarities in induced gene expression among LPS and MPLA were further illustrated using networks and functions analysis. The Molecule Activity Predictor (MAP) tool in IPA enables one to simulate the upstream and/or downstream effects of activation or inhibition of molecules in a network given a starting set of neighboring molecules with “known” activity or expression. In both groups, the top predicted network is centered on tumor necrosis factor-alpha (TNF-α).

The functions results of the IPA core analysis not only allowed transcripts to be grouped under particular functional annotations, but were also used to determine which functions were likely increased or decreased by integrating the direction of the fold change of a particular molecule and its documented impact on that function in the literature. In our study, among all the functions that were predicted to have significantly increased activity in both LPS and MPLA groups, only 5 functions were found distinct in each group and the inflammatory response and hematological system development were the shared top two predicted functions in the two groups.

In an effort to identify other potential drugs bearing immunomodulatory traits that are similar to LPS or MPLA, we used LPS- and MPLA-responsive genes and Ingenuity upstream regulator analysis to identify the upstream regulators that may cause similar gene expression changes compared to those induced by LPS and MPLA. Upstream regulators are defined as any molecule that can affect the expression of another molecule, including transcription factors, cytokines, drugs, and chemicals. Receptors and molecules that are known to induce the activation of inflammatory genes were found among the predicted activated upstream regulators for both compounds and had nearly complete overlap. The inhibitor analysis demonstrated complete overlap when comparing groups and indicates that LPS and MPLA activate MAP kinase and phosphoinositide-3-kinase signaling.

There are limitations to our study. We did not perform FACS analysis to characterize the composition of the immune cells used in our array due to the known technical difficulties associated with performing the red blood cell lysis procedure. However, existing literatures have shown that, (1) the main targets of the LPS/MPLA treatment in human blood are monocytes and neutrophils^33^; and (2) Toll-like receptor 4 (TLR4), the essential receptor for LPS recognition, is mainly expressed by monocytes (and neutrophils but at lower levels) in human peripheral blood^34^. In addition, in our study, the 90-minute incubation period is not long enough for cells to proliferate. Thus, the composition of leukocytes in the samples is unlikely to change during the treatment period.

On the other hand, our transcriptome analysis was based on the RNA extracted from the whole blood, not from the enriched peripheral blood mononuclear cell (PBMCs) fraction. Nevertheless, we validated many of the key findings using isolated peripheral blood mononuclear cells and extended observations at the transcriptional level by demonstrating parallel production and secretion of protein products. Yet, analysis of blood only provides an assessment of one organ system and may not represent the transcriptional response elicited in other tissues. However, the peripheral blood provides a rich source of leukocytes and is the only tissue readily available for harvest in humans. Our findings validate, in a human system, previous observations made in mouse models. The use of human systems is important for further development of TLR4 agonists for use in humans and supported by the success of MPLA as a vaccine adjuvant in humans.

LPS is known for its toxicity in humans, which precludes its clinical use; while MPLA, a less toxic derivative of LPS, is currently employed as an adjuvant in marketed vaccines. We have published results showing that MPLA is effective in broadly enhancing innate immune responses against multiple clinically relevant bacterial pathogens^20^,^21^,^35^. As we move our studies forward, it is valuable to know the transcriptional response of human leukocytes to LPS and MPLA since the information will provide important insights to guide future mechanistic studies and characterize important similarities and differences in the transcriptional response to the two agents. In addition, our present study provides critical results to further advance our knowledge with respect to key differences in the molecular pathways that are differentially regulated by MPLA and LPS in humans PBMCs. This further enables to elucidate the molecular targets responsible for the preferential low toxicity of MPLA as compared to LPS. Finally, many previous studies were performed in mice. The present study advances knowledge regarding the transcriptional response in humans.

## Material and Methods

### Reagents and Dosing

Both LPS (Catalog number: L2630) and MPLA (Catalog number: L6895) were purchased from Sigma-Aldrich Corp. (St. Louis, MO). LPS was prepared according to the manufacturer’s instructions. MPLA was solubilized in 0.2% triethylamine solution (1 mg/ml) and sonicated for 1 hour at 40 °C. The doses for LPS and MPLA used in this study were 1 μg/ml and 10 μg/ml, respectively, and were determined based on dose response curves using TNFα and IFNγ secretion as the endpoint. The doses chosen were based on the plateau of maximal TNFα and IFNγ production in response to each agent.

### Blood Sample Preparation

All participants of this study signed an informed consent along with a trained team member. The consent procedure and experimental protocol were approved by the Institutional Review Board (IRB Number: 131331) of Vanderbilt University Medical Center. All methods were performed in accordance with the relevant guidelines and regulations. After obtaining written consents, blood was drawn from six healthy human adult volunteers, and collected in tubes containing lithium heparin. All blood samples were prepared for incubation within 10 minutes of blood draw in order to ensure the quality of the cells used in microarray analysis. Three mL of blood sample was incubated at 37 °C for 90 minutes on a rolling rocker with 30 μl of either PBS, LPS or MPLA with corresponding doses. Then the blood was transferred to PAXgene Blood RNA tubes and incubated for another 8 hours at room temperature on a rolling rocker. PAXgene Blood RNA tubes help in efficient stabilization of RNA till subsequent processing for isolation and purification of RNA from whole blood. Samples were stored in the freezer at −20 °C until shipped to GenUs BioSystems (Northbrook, IL) for the microarray assay.

### Total RNA Extraction and Quantification

Under an RNase-free environment, human blood total RNA was isolated using a Ribopure RNA isolation kit (Thermo Fisher Scientific, Waltham, MA). RNA concentration and quality were verified with an Agilent Bioanalyzer (Agilent Tecknologies, Santa Clara, CA), using RNA6000 Nano reagents (Agilent Technologies, Santa Clara, CA). Purified RNA was used for quantitative Real-Time PCR in our laboratory and for microarray assays performed at GenUs BioSystems (Northbrook, IL).

### cRNA Preparation for Microarray Assay

After high-fidelity linear amplification of the Poly(A) + RNA population, human blood total RNA was reverse transcribed using RiboAmp HS^Plus^ RNA Amplification Kit (Life Technologies Inc., Burlington, ON) by priming with a DNA oligonucleotide containing T7 RNA polymerase promoter 5′ to a d(T)24 sequence. Thereafter, the second-strand cDNA and double-stranded cDNA was synthesized and purified immediately. Furthermore, the cDNA serves as the template in an *in vitro* transcription (IVT) reaction to produce cRNA using T7 RNA polymerase. The IVT is performed in the presence of biotinylated nucleotides to label the target cRNA. The quantity and quality of the labeled cRNA was assayed by spectrophotometry on an Agilent Bioanalyzer.

### Microarray Processing Performed by GenUs BioSystems

Briefly, 1 μg of purified cRNA was fragmented at 60 °C for 30 minutes in fragmentation buffer to uniform size. On completion of the fragmentation reaction, hybridization was carried out with Agilent Human 8 × 60 K v2 Gene Expression microarrays (Agilent Technologies, Human design ID 039494) for 18 hours at 37 °C in a rotating Agilent hybridization oven. After Hybridization, microarrays were washed for 1 minute at room temperature with GE Wash Buffer 1 (Agilent Technologies, Santa Clara, CA) and 1 minute at 37 °C with GE Wash Buffer 2 (Agilent Technologies, Santa Clara, CA), then dried immediately by brief centrifugation. Arrays were scanned on a G2565 Microarray Scanner at 5 μM resolution (Agilent Technologies, Santa Clara, CA). Agilent Feature Extraction Software 10.7 was used to process the scanned images from arrays using default parameters (gridding and feature intensity extraction) to obtain background subtracted and spatially detrended processed intensities. Feature Non-uniform outliers shown in “Feature Extraction” were excluded. Data generated for each probe on the array were normalized using quantile normalization in R statistical language.

### Quantitative Real-Time PCR

Isolated human blood total RNA was reverse transcribed with the iScript cDNA Synthesis Kit (Cat. # 1708890, Bio-Rad Laboratories), and mRNA expression was analyzed by Real-Time PCR with the SsoFast EvaGreen Supermix Kit (Cat. # 1725201, Bio-Rad Laboratories). Real-Time PCR reactions were run in triplicate on a CFX96 Touch Real-Time PCR Detection System (Bio-Rad Laboratories) and quantification of gene expression was determined by the comparative ΔΔCt method. The mRNA expression was normalized to the endogenous reference gene *HPRT1* (hypoxanthine phosphoribosyl transferase 1). *RPLP0* (Ribosomal Protein, Large, P0) was also used in our study as an additional reference gene for qPCR analysis, but there was no difference in the data generated with it as compared to that with *HPRT1*. Primers for the genes analyzed are listed in Table 1.

### Western Blotting

Western Blot analysis was performed to determine the protein levels of inflammasome components. In these studies, human PBMCs were isolated from whole blood with Lymphoprep, (Ref. no. 1114545; Axis-Shield, Oslo, Norway), and then incubated with PBS, MPLA, or LPS at 37 °C for 6 hours. PBMCs were disrupted and lysed in cold RIPA lysis buffer (R0278, Sigma-Aldrich) containing 2% Complete EDTA-free Protease Inhibitor Cocktail (04693132001, Roche Applied Science), and then heated for 5 minutes at 95 °C and spun for 15 minutes at 15,000 g to remove insoluble material. Protein (25 μg per well) was separated on 4–20% gradient Mini-PROTEIN TGX Precast Gels (#456-1094, Bio-Rad Laboratories) and transferred to a nitrocellulose membrane (162-0145, Bio-Rad Laboratories). The membrane was blocked in TBS-T (0.1% Tween20 in Tris-Buffered Saline buffer) with 5% BSA (A30075, RPI Corp., Mount Prospect, IL)) for 1 hour at room temperature, and then incubated over-night with specific antibodies against NLRP3 (110 kDa; 1:1,000; #15101; Cell Signaling Technology), Caspase-1 (45 kDa; 1:200; sc-515; Santa Cruz), IL-1B (31 kDa; 1:1,000; #12703; Cell Signaling Technology), IL-18 (24 kDa; 1:200; sc-7954; Santa Cruz), or beta-Actin (42 kDa; 1:20,000; A2228; Sigma-Aldrich). Matching horseradish peroxidase-conjugated secondary antibody was applied at 1:5,000 and immunoreactive protein bands were detected using Western Lightning Plus-ECL (NEL103001EA, PerkinElmer). Western blot quantification was performed using the NIH ImageJ software and normalized to beta-Actin.

### Measurement of Cytokine Secretion

Human blood samples were incubated with PBS, MPLA, or LPS at 37 °C for 6 hours and 24 hours, and then the supernatant was harvested for cytokine production measurement. Interferon beta-1 (IFNB1) (41415, PBL Assay Science), IL-23 (88-7237 eBioscience), and CCL7 (88-50700, eBioscience) concentrations were measured using ELISA according to the manufacturer’s protocols. Cytokine concentrations were determined by measuring optical density at 450 nm using a microtiter plate reader (Dynatech Laboratories, Chantilly, VA, USA). Concentrations of IL-6, IL-1α, TNF-α, IL-8, IL-12B, IFN-γ, CXCL10, IL-1β, and IL-18 were measured by use of a customized Bio-Plex Multiplex Assay and MAGPIX Multiplex Reader (Bio-Rad Laboratories). Results were analyzed with Bio-Plex Manager Software 6.1, and graphs were made with GraphPad Prism Software 6.0 (GraphPad Software, San Diego, CA, USA).

### Ingenuity Pathway Analysis (IPA) and Statistical Tests

Obtained datasets were filtered by using cut-off values of 0.1 and 2 for the false discovery rate (FDR) and fold change, respectively, to identify the differentially expressed genes after LPS or MPLA treatment. After uploading these differentially expressed genes and their corresponding expression values into the Ingenuity Pathway Analysis (IPA) software (Ingenuity Systems, Redwood City, CA), a “core analysis” was performed separately for each group, using default parameters. Each gene symbol was mapped to its corresponding gene object in the Ingenuity Pathways Knowledge Database and then overlaid onto a global molecular network to algorithmically generate networks of these genes based on their connectivity. These computationally generated networks indicate the biological relationships among genes.

In addition, “Canonical Pathways Analysis” was performed to determine genes that were involved in well-documented canonical signal transduction or metabolic pathways, from the library of canonical pathways in Ingenuity Pathways Knowledge Database. Benjamini–Hochberg procedure for multiple testing corrections, which allows us to calculate the false discovery rate for each of the probability values, determines the probability that the association between the genes in our datasets and the canonical pathway is not explained by chance alone. A ratio of the number of genes from our datasets that map to the pathway divided by the total number of genes that exist within the canonical pathway determines the significance of the association between the molecules in our datasets and identified canonical pathway.

“Diseases and Functions” analysis was used to group transcripts under particular functional annotations, and hence to identify the biological functions that were likely increased or decreased based on the direction of the fold change of a particular gene and its impact on that function documented in Ingenuity Pathways Knowledge Database. IPA program generates an activation “z-score” for each functional category, which indicates the effects to the biological process are trending towards an increase (a positive z-score) or a decrease (a negative z-score). This provides a prediction of activation or inhibition of each function annotation. Z-scores ≥2 or ≤−2 indicates that the function’s trend is statistically significant.

Furthermore, “Upstream Regulators” analysis section of the core analysis was used to identify the cascade of upstream transcriptional regulators that were involved in each treatment and whether they were likely activated or inhibited to obtain the observed gene expression profile changes in our datasets. This transcription regulator prediction can help to provide a testable hypothesis by explaining how these upstream molecules and their targets may regulate the altered biological processes, pathways, and functions.

### Cytokine, PCR and Western Blot Statistical Tests

Results were tested for statistical significance using one-way ANOVA and Tukey post-hoc test to correct for multiple comparisons among PBS, MPLA, and LPS groups. All data calculations and graph preparations were performed using GraphPad Prism 6.0 (GraphPad Software Inc., San Diego, CA). All data values are presented as mean ± SEM. *p < 0.05, **p < 0.01, ***p < 0.001, ****p < 0.0001, NS = not significant.

## Additional Information

**How to cite this article**: Luan, L. *et al*. Comparative Transcriptome Profiles of Human Blood in Response to the Toll-like Receptor 4 Ligands Lipopolysaccharide and Monophosphoryl Lipid A. *Sci. Rep.* **7**, 40050; doi: 10.1038/srep40050 (2017).

**Publisher's note:** Springer Nature remains neutral with regard to jurisdictional claims in published maps and institutional affiliations.

## Supplementary Material

Supplementary Dataset 1

## Figures and Tables

**Table 1:** Primer Sequences Used for Real-Time PCR.

	Gene Name	GenBank Accession number	5′ primer	3′ primer
Up-regulated genes	*IL6*	NM_000600	CCACTCACCTCTTCAGAACG	CATCTTTGGAAGGTTCAGGTTG
	*CCL20*	NM_004591	GGTGAAATATATTGTGCGTCTCC	ACTAAACCCTCCATGATGTGC
	*CXCL3*	NM_002090	AAGTGTGAATGTAAGGTCCCC	GTGCTCCCCTTGTTCAGTATC
	*IL1A*	NM_000575	TGTATGTGACTGCCCAAGATG	TTAGTGCCGTGAGTTTCCC
	*TNF*α	NM_000594	ACTTTGGAGTGATCGGCC	GCTTGAGGGTTTGCTACAAC
	*CCL3L3*	NM_001001437	AATCATGCAGGTCTCCACTG	GAATCTGTCGGGAGGTGTAG
Down-regulated genes	*RHOB*	NM_004040	AGAACGGCTGCATCAACTG	CTTGTGGGACACGGGTC
	*TMEM170B*	NM_001100829	AGTGTTGATGTTTGTGATGCTG	CTACTCTGTAAATGCCCGCTAC
	*CCR2*	NM_001123041	ACTCACTGCTGCATCAATCC	CATTCTTTCCTGGTCTCACTCC
	*KCNE3*	NM_005472	TCCAGAGACATCCTGAAGAGG	TCTCCATAGCAACAGGGATTG
	*FRAT2*	NM_012083	CTCAGGCTCCTTGCTCTG	CGGCTTCAGCTCAGAGTTAG
	*MYCL1*	NM_001033081	GCGAACCCAAGACCCAG	CTTCCGAATACCCAGAGACTG
Non-regulated genes	*JOSD1*	NM_014876	CCTCCACGCCCTCAATAAC	TCGTAGTTGCCATTTCCCAG
	*NAGS*	NM_153006	TCTTCCTCAATAACACAGGCG	GTTCTTTTGTGCTCACCCAC
Other genes of interest	*IFNB1*	NM_002176	GCCAAGGAGTACAGTCACTG	TGAAGCAATTGTCCAGTCCC
	*IL23A*	NM_016584	ATGTTCCCCATATCCAGTGTG	GCTCCCCTGTGAAAATATCCG
	*CCL7*	NM_006273	GAGAGCTACAGAAGGACCAC	GTTTTCTTGTCCAGGTGCTTC
	*TNFAIP2*	NM_006291	GGAGCAGAATTGGCAGGTAC	TGCGTGAACCTCTTGAACAG
	*IL1B*	NM_000576	ATGCACCTGTACGATCACTG	ACAAAGGACATGGAGAACACC
	*IL8*	NM_000584	ATACTCCAAACCTTTCCACCC	TCTGCACCCAGTTTTCCTTG
	*IL12B*	NM_002187	CACATTCCTACTTCTCCCTGAC	CTGAGGTCTTGTCCGTGAAG
	*IL18*	NM_001562	CATTGACCAAGGAAATCGGC	CACAGAGATAGTTACAGCCATACC
	*IFNG*	NM_000619	GCATCGTTTTGGGTTCTCTTG	AGTTCCATTATCCGCTACATCTG
	*CXCL10*	NM_001565	CCTTATCTTTCTGACTCTAAGTGGC	ACGTGGACAAAATTGGCTTG
Reference genes	*HPRT1*	NM_000194	TGGCGTCGTGATTAGTGATG	ACCCTTTCCAAATCCTCAGC
	*RPLP0*	NM_001002	TCGACAATGGCAGCATCTAC	GCCTTGACCTTTTCAGCAAG

The 5′ and 3′ primers, along with corresponding GenBank Accession numbers, used for the up-regulated, down-regulated, non-regulated, and other genes of interest that were evaluated by qPCR are shown.
