# Supplementary material for: Comparative Transcriptome Profiles of Human Blood in Response to the Toll-like Receptor 4 Ligands Lipopolysaccharide and Monophosphoryl Lipid A
Source: Sci Rep. 2017 Jan 5;7:40050. doi: 10.1038/srep40050 (PMC5215261; doi:10.1038/srep40050)
Supplement: Supplementary Dataset 1 [file srep40050-s1.doc]

**Comparative Transcriptome Profiles of Human Blood in Response to the Toll-like Receptor 4 Ligands Lipopolysaccharide and Monophosphoryl Lipid A**

Liming Luan^1*^, Naeem K.Patil^1^, Yin Guo^2^, Antonio Hernandez^1^, Julia K. Bohannon^1^, Benjamin A. Fensterheim^2^, Jingbin Wang^1^, Yaomin Xu^3^, Perenlei Enkhbaatar^4^, Ryan Stark^5^, and Edward R. Sherwood^1,2*^

**Supplemental data**

**S1: Lists of common genes affected by both LPS and MPLA**

| **Microarray results** | **Genes** | **Total number** |
| --- | --- | --- |
| **Commonly up-regulated genes** | ACHE, ARL5B, C11orf96, C15orf48, CCL20, CCL3, CCL3L3, CCL4, CCRL2, CD69, CD83, CLIC4, CXCL1, CXCL2, CXCL3, DCUN1D3, DUSP5, EDN1, EMX1, F3, FGD4, FOSB, FXYD3, G0S2, GABPB1, GADD45B, GCH1, GJB2, GPR84, GRASPOS, ICAM1, IER3, IFNB1, IFNG, IL18, IL1A, IL1B, IL1RN, IL36G, IL6, IRAK2, IRG1, KCNK15, KMO, KRTAP5-11, KYNU, LAMB3, LINC00346, MAPK6, MFSD2A, NEAT1, NFKB1, NFKBIA, NFKBIE, NFKBIZ, NLRP3, PFKFB3, PI3, PIM3, PLAU, PLEK, PPAN, PTX3, RASGEF1B, RIPK2, SERPINB2, SLAMF7, SLC2A6, TAGAP, TH, TNF, TNFAIP2, TNFAIP3, ZFP36 | 74 |
| **Commonly down-regulated genes** | FRAT2, KCNE3, KCTD12, MYCL1, PPP1R3B, RHOB, TLR1, TMEM170B, TRIM25 | 9 |

**S2: Lists of genes affected by LPS alone**

| **Microarray results** | **Genes** | **Total number** |
| --- | --- | --- |
| **LPS uniquely up-regulated genes** | BCL2A1, C19orf66, CCL23, CERS3, CFLAR, CXCL10, DNAJA1 , DRAM1, EBI3, EIF5AL1, EREG, FLJ36644, FSCN1, GPR183, IER5, IL12B, IL23A, IL28A, IL4, KCNJ2, MAP3K8, MIR155HG, ORM2, OTUD1, PDE4B, PLK2, PPP1R17, PTGER2, PTGER4, RILPL2, RNF144B, RNU4ATAC, SAMSN1, SCARNA17, SCARNA9, SOCS3, TNFAIP6, TNFSF9, TNIP3, TRAF1, XAGE1A | 41 |
| **LPS uniquely down-regulated genes** | ANKRD58, ARHGEF40, CCR2, CENPBD1, FADD, HMOX1, PELO, PHF23, S1PR3, THBD, TNFRSF1A, TNFRSF8 | 12 |

**S3: Lists of genes affected by MPLA alone**

| **Microarray results** | **Genes** | **Total number** |
| --- | --- | --- |
| **MPLA uniquely up-regulated genes** | AK4, BHLHE40, CCL2, CCL7, CCR5, CH25H, CSRNP1, CTSL1, CYB5D1, DUSP1, EGR3, EHD4, ETF1, IL10, IL4I1, IL8, INHBA, ITPRIP, KLF10, LHFPL2, MAFB, MPDU1, NR4A3, NRARP, NSMAF, OLIG1, PHACTR1, PLIN2, PNPLA8, PPIF, RAB7B, RABGEF1, SGK1, SPINK1, TBC1D7, TICAM1, TRIB3, TUFT1, ZC3H12C | 39 |
| **MPLA uniquely down-regulated genes** | CX3CR1, FRAT1, IL17RA, LMO2, NCEH1, NUAK2, SAP30L, ST8SIA4 | 8 |

**S4: Total LPS up-regulated genes (115 genes)**

| **Gene** | **Fold Change** | **P.Value** |
| --- | --- | --- |
| IL6 | 148.10 | 2.27E-13 |
| CCL20 | 61.80 | 1.32E-12 |
| CXCL3 | 44.34 | 1.35E-13 |
| IL1A | 44.04 | 2.73E-11 |
| CCL3L3 | 29.48 | 8.61E-08 |
| PTX3 | 27.02 | 7.79E-12 |
| IFNB1 | 26.17 | 2.33E-08 |
| GPR84 | 25.69 | 7.33E-10 |
| C15orf48 | 23.99 | 1.34E-09 |
| IRG1 | 21.05 | 5.83E-08 |
| CXCL2 | 17.00 | 9.87E-09 |
| KRTAP5-11 | 16.24 | 7.31E-11 |
| CCL4 | 14.38 | 7.42E-13 |
| EMX1 | 12.48 | 9.95E-10 |
| IL1RN | 11.95 | 1.47E-10 |
| CCL3 | 10.84 | 6.46E-10 |
| TNFAIP3 | 10.22 | 2.00E-08 |
| NLRP3 | 9.98 | 5.45E-08 |
| TNF | 9.60 | 3.00E-11 |
| F3 | 9.16 | 7.58E-08 |
| CCRL2 | 8.67 | 1.78E-08 |
| CLIC4 | 8.65 | 3.39E-05 |
| CD83 | 8.48 | 6.33E-09 |
| IL1B | 8.24 | 1.81E-08 |
| NFKBIZ | 8.18 | 9.83E-06 |
| FXYD3 | 8.03 | 1.49E-09 |
| CXCL10 | 7.49 | 1.22E-04 |
| TNFAIP6 | 7.28 | 1.22E-04 |
| IFNG | 6.87 | 5.64E-05 |
| KYNU | 6.87 | 9.44E-07 |
| PLAU | 6.84 | 1.21E-04 |
| ICAM1 | 6.28 | 2.10E-08 |
| EREG | 6.23 | 1.61E-04 |
| ARL5B | 5.97 | 3.75E-06 |
| GRASPOS | 5.97 | 2.17E-05 |
| IER3 | 5.66 | 6.10E-05 |
| SERPINB2 | 5.63 | 2.40E-05 |
| IL12B | 5.46 | 5.50E-06 |
| KCNJ2 | 5.08 | 1.53E-05 |
| IL23A | 5.08 | 4.19E-08 |
| PLK2 | 5.03 | 1.31E-04 |
| ACHE | 4.80 | 3.92E-07 |
| TNFAIP2 | 4.79 | 6.04E-08 |
| NFKBIA | 4.70 | 2.28E-09 |
| KMO | 4.65 | 4.16E-05 |
| PLEK | 4.44 | 1.18E-05 |
| BCL2A1 | 4.41 | 2.79E-04 |
| IRAK2 | 4.36 | 6.62E-04 |
| MFSD2A | 4.29 | 3.79E-06 |
| RIPK2 | 4.26 | 3.09E-06 |
| PPP1R17 | 4.18 | 8.61E-05 |
| IL4 | 4.07 | 7.33E-05 |
| SLAMF7 | 3.97 | 2.39E-05 |
| IL18 | 3.96 | 7.93E-07 |
| FLJ36644 | 3.96 | 1.29E-04 |
| SAMSN1 | 3.95 | 7.04E-05 |
| LAMB3 | 3.93 | 7.10E-05 |
| DUSP5 | 3.75 | 9.83E-06 |
| SLC2A6 | 3.67 | 9.46E-07 |
| XAGE1A | 3.64 | 1.01E-04 |
| TAGAP | 3.61 | 7.70E-05 |
| CXCL1 | 3.60 | 1.82E-04 |
| MAP3K8 | 3.60 | 1.77E-06 |
| DRAM1 | 3.57 | 3.02E-05 |
| EIF5AL1 | 3.55 | 8.11E-04 |
| CD69 | 3.51 | 3.53E-06 |
| DCUN1D3 | 3.50 | 1.39E-05 |
| GCH1 | 3.49 | 2.10E-04 |
| GPR183 | 3.42 | 1.45E-04 |
| G0S2 | 3.40 | 9.07E-04 |
| RNF144B | 3.34 | 1.29E-03 |
| NFKBIE | 3.31 | 1.88E-05 |
| LINC00346 | 3.24 | 1.19E-04 |
| GADD45B | 3.23 | 9.10E-06 |
| CFLAR | 3.22 | 1.69E-06 |
| MIR155HG | 3.15 | 5.44E-04 |
| KCNK15 | 3.14 | 1.62E-04 |
| PFKFB3 | 3.09 | 3.66E-04 |
| TRAF1 | 3.04 | 2.59E-04 |
| IER5 | 3.02 | 6.38E-06 |
| CCL23 | 2.99 | 9.25E-04 |
| PI3 | 2.94 | 1.14E-04 |
| CERS3 | 2.92 | 7.38E-04 |
| C11orf96 | 2.90 | 2.29E-04 |
| TH | 2.82 | 8.16E-05 |
| FGD4 | 2.81 | 3.97E-04 |
| EBI3 | 2.78 | 1.01E-05 |
| ORM2 | 2.78 | 4.85E-04 |
| GJB2 | 2.76 | 1.00E-03 |
| SCARNA9 | 2.75 | 3.52E-05 |
| PTGER4 | 2.75 | 1.27E-03 |
| SCARNA17 | 2.74 | 4.29E-04 |
| PIM3 | 2.73 | 1.35E-05 |
| FSCN1 | 2.73 | 1.15E-03 |
| ZFP36 | 2.72 | 3.43E-04 |
| PPAN | 2.68 | 2.37E-04 |
| C19orf66 | 2.62 | 1.61E-04 |
| IL28A | 2.57 | 8.09E-04 |
| PDE4B | 2.56 | 4.88E-05 |
| NEAT1 | 2.51 | 1.48E-05 |
| DNAJA1 | 2.49 | 3.70E-04 |
| TNIP3 | 2.43 | 4.01E-04 |
| TNFSF9 | 2.42 | 3.05E-04 |
| MAPK6 | 2.41 | 8.42E-04 |
| RASGEF1B | 2.38 | 2.14E-04 |
| RILPL2 | 2.36 | 7.02E-05 |
| RNU4ATAC | 2.33 | 3.35E-04 |
| GABPB1 | 2.31 | 9.11E-04 |
| PTGER2 | 2.29 | 6.71E-04 |
| SOCS3 | 2.29 | 1.14E-03 |
| FOSB | 2.27 | 1.82E-04 |
| NFKB1 | 2.26 | 9.67E-04 |
| OTUD1 | 2.22 | 1.35E-04 |
| IL36G | 2.14 | 5.75E-04 |
| EDN1 | 2.06 | 6.80E-04 |

**S5: Total LPS down-regulated genes (21 genes)**

| **Gene** | **Fold Change** | **P.Value** |
| --- | --- | --- |
| RHOB | 0.14 | 3.84E-10 |
| TMEM170B | 0.24 | 8.97E-06 |
| CCR2 | 0.24 | 4.39E-04 |
| KCNE3 | 0.25 | 6.20E-06 |
| FRAT2 | 0.25 | 6.77E-06 |
| PPP1R3B | 0.26 | 1.25E-06 |
| THBD | 0.29 | 1.49E-05 |
| MYCL1 | 0.31 | 2.80E-05 |
| S1PR3 | 0.31 | 1.38E-04 |
| ARHGEF40 | 0.31 | 1.94E-05 |
| TLR1 | 0.36 | 5.71E-04 |
| TRIM25 | 0.36 | 6.32E-05 |
| PHF23 | 0.37 | 4.99E-04 |
| HMOX1 | 0.38 | 2.45E-04 |
| KCTD12 | 0.38 | 5.49E-04 |
| TNFRSF8 | 0.38 | 2.94E-05 |
| ANKRD58 | 0.39 | 7.75E-04 |
| PELO | 0.40 | 1.12E-05 |
| TNFRSF1A | 0.46 | 1.19E-03 |
| CENPBD1 | 0.48 | 2.05E-04 |
| FADD | 0.48 | 7.91E-04 |

**S6: Total MPLA up-regulated genes (113 genes)**

| **Gene** | **Fold Change** | **P.Value** |
| --- | --- | --- |
| IL6 | 43.35 | 3.93E-11 |
| C15orf48 | 30.56 | 3.80E-10 |
| PTX3 | 28.86 | 5.44E-12 |
| CCL3L3 | 22.16 | 8.37E-09 |
| IL1A | 18.51 | 5.31E-10 |
| CCL20 | 17.66 | 8.37E-10 |
| CXCL3 | 16.95 | 2.87E-11 |
| PLAU | 16.15 | 2.67E-07 |
| CCL7 | 14.33 | 9.40E-08 |
| CXCL2 | 12.53 | 6.12E-08 |
| F3 | 12.12 | 1.14E-08 |
| CCRL2 | 11.73 | 2.06E-09 |
| CCL4 | 11.54 | 3.61E-12 |
| GPR84 | 11.30 | 8.71E-08 |
| IRG1 | 11.19 | 1.84E-06 |
| CCL3 | 10.08 | 1.10E-09 |
| KRTAP5-11 | 9.30 | 3.41E-09 |
| NFKBIZ | 8.42 | 8.22E-06 |
| SERPINB2 | 8.03 | 2.08E-06 |
| EMX1 | 7.93 | 2.65E-08 |
| IER3 | 7.33 | 1.12E-05 |
| IL8 | 7.26 | 2.15E-05 |
| KMO | 7.12 | 1.76E-06 |
| CXCL1 | 7.04 | 2.41E-04 |
| TNF | 6.91 | 4.72E-10 |
| CD83 | 6.80 | 3.71E-08 |
| LAMB3 | 6.55 | 1.19E-06 |
| TNFAIP3 | 5.89 | 1.23E-06 |
| DUSP5 | 5.80 | 1.76E-07 |
| FXYD3 | 5.72 | 2.80E-08 |
| IL1B | 5.64 | 6.34E-08 |
| ICAM1 | 5.36 | 6.78E-08 |
| SPINK1 | 5.27 | 4.71E-05 |
| CCL2 | 4.95 | 1.67E-04 |
| IL1RN | 4.94 | 2.06E-07 |
| IRAK2 | 4.86 | 3.28E-04 |
| KYNU | 4.86 | 1.37E-05 |
| NLRP3 | 4.79 | 1.32E-05 |
| C11orf96 | 4.64 | 2.74E-06 |
| IFNB1 | 4.52 | 5.47E-04 |
| GJB2 | 4.52 | 5.38E-06 |
| NFKBIA | 4.46 | 1.08E-08 |
| ZC3H12C | 4.29 | 1.26E-03 |
| DCUN1D3 | 4.23 | 2.09E-06 |
| G0S2 | 4.22 | 1.83E-04 |
| IFNG | 4.19 | 1.16E-03 |
| INHBA | 4.16 | 1.57E-05 |
| TAGAP | 4.09 | 2.61E-05 |
| PPIF | 4.08 | 5.80E-05 |
| PHACTR1 | 4.01 | 1.74E-06 |
| CLIC4 | 3.87 | 1.36E-04 |
| CD69 | 3.85 | 5.24E-06 |
| CH25H | 3.76 | 3.04E-04 |
| EDN1 | 3.71 | 5.77E-05 |
| NR4A3 | 3.64 | 8.55E-04 |
| GRASPOS | 3.60 | 7.83E-04 |
| TH | 3.55 | 7.02E-06 |
| CTSL1 | 3.54 | 1.69E-04 |
| SLAMF7 | 3.50 | 7.44E-05 |
| OLIG1 | 3.50 | 6.45E-06 |
| EGR3 | 3.44 | 1.08E-04 |
| ACHE | 3.40 | 1.19E-05 |
| ARL5B | 3.33 | 3.93E-04 |
| LINC00346 | 3.31 | 9.88E-05 |
| SLC2A6 | 3.29 | 3.21E-06 |
| KLF10 | 3.27 | 1.23E-04 |
| DUSP1 | 3.27 | 2.10E-04 |
| TUFT1 | 3.21 | 1.68E-04 |
| RABGEF1 | 3.18 | 3.87E-04 |
| TRIB3 | 3.09 | 4.82E-04 |
| MFSD2A | 3.07 | 9.71E-05 |
| SGK1 | 3.01 | 1.01E-04 |
| PLIN2 | 3.00 | 3.22E-04 |
| NEAT1 | 3.00 | 1.38E-06 |
| FGD4 | 2.96 | 2.42E-04 |
| MAPK6 | 2.95 | 1.07E-04 |
| PLEK | 2.92 | 4.87E-04 |
| PFKFB3 | 2.90 | 6.35E-04 |
| CSRNP1 | 2.89 | 4.72E-04 |
| ZFP36 | 2.85 | 2.18E-04 |
| GADD45B | 2.80 | 4.48E-05 |
| GCH1 | 2.77 | 3.87E-04 |
| MPDU1 | 2.77 | 6.94E-04 |
| LHFPL2 | 2.72 | 1.08E-03 |
| RIPK2 | 2.60 | 4.19E-04 |
| TICAM1 | 2.60 | 8.45E-04 |
| NFKBIE | 2.58 | 2.60E-04 |
| NSMAF | 2.58 | 3.20E-04 |
| MAFB | 2.58 | 8.56E-04 |
| AK4 | 2.57 | 4.20E-04 |
| CCR5 | 2.56 | 3.92E-05 |
| CYB5D1 | 2.52 | 2.62E-04 |
| KCNK15 | 2.49 | 1.21E-03 |
| RASGEF1B | 2.49 | 1.26E-04 |
| TBC1D7 | 2.42 | 7.15E-05 |
| EHD4 | 2.41 | 2.93E-04 |
| IL4I1 | 2.40 | 9.12E-04 |
| NFKB1 | 2.39 | 2.92E-04 |
| IL36G | 2.38 | 1.54E-04 |
| PPAN | 2.38 | 8.39E-04 |
| PIM3 | 2.37 | 8.23E-05 |
| PI3 | 2.34 | 1.19E-03 |
| ITPRIP | 2.32 | 9.93E-05 |
| IL10 | 2.31 | 1.28E-03 |
| BHLHE40 | 2.30 | 1.15E-04 |
| GABPB1 | 2.30 | 9.84E-04 |
| IL18 | 2.12 | 1.02E-03 |
| TNFAIP2 | 2.10 | 7.68E-04 |
| PNPLA8 | 2.08 | 1.31E-03 |
| RAB7B | 2.08 | 6.03E-04 |
| NRARP | 2.05 | 6.52E-04 |
| ETF1 | 2.02 | 5.85E-04 |
| FOSB | 2.01 | 8.66E-04 |

**S7: Total MPLA down-regulated genes (17 genes)**

| **Gene** | **Fold Change** | **P.Value** |
| --- | --- | --- |
| KCNE3 | 0.20 | 9.65E-07 |
| RHOB | 0.26 | 1.49E-07 |
| TMEM170B | 0.27 | 2.75E-05 |
| PPP1R3B | 0.28 | 3.54E-06 |
| MYCL1 | 0.29 | 1.42E-05 |
| FRAT2 | 0.29 | 3.03E-05 |
| NUAK2 | 0.34 | 6.73E-04 |
| IL17RA | 0.36 | 4.97E-04 |
| TLR1 | 0.37 | 6.26E-04 |
| KCTD12 | 0.37 | 4.12E-04 |
| FRAT1 | 0.38 | 2.90E-04 |
| ST8SIA4 | 0.44 | 7.01E-04 |
| TRIM25 | 0.45 | 7.59E-04 |
| CX3CR1 | 0.46 | 2.27E-04 |
| SAP30L | 0.46 | 9.97E-05 |
| LMO2 | 0.47 | 1.37E-03 |
| NCEH1 | 0.48 | 1.13E-03 |

**S8: Full-length gel images of Figure 6C**


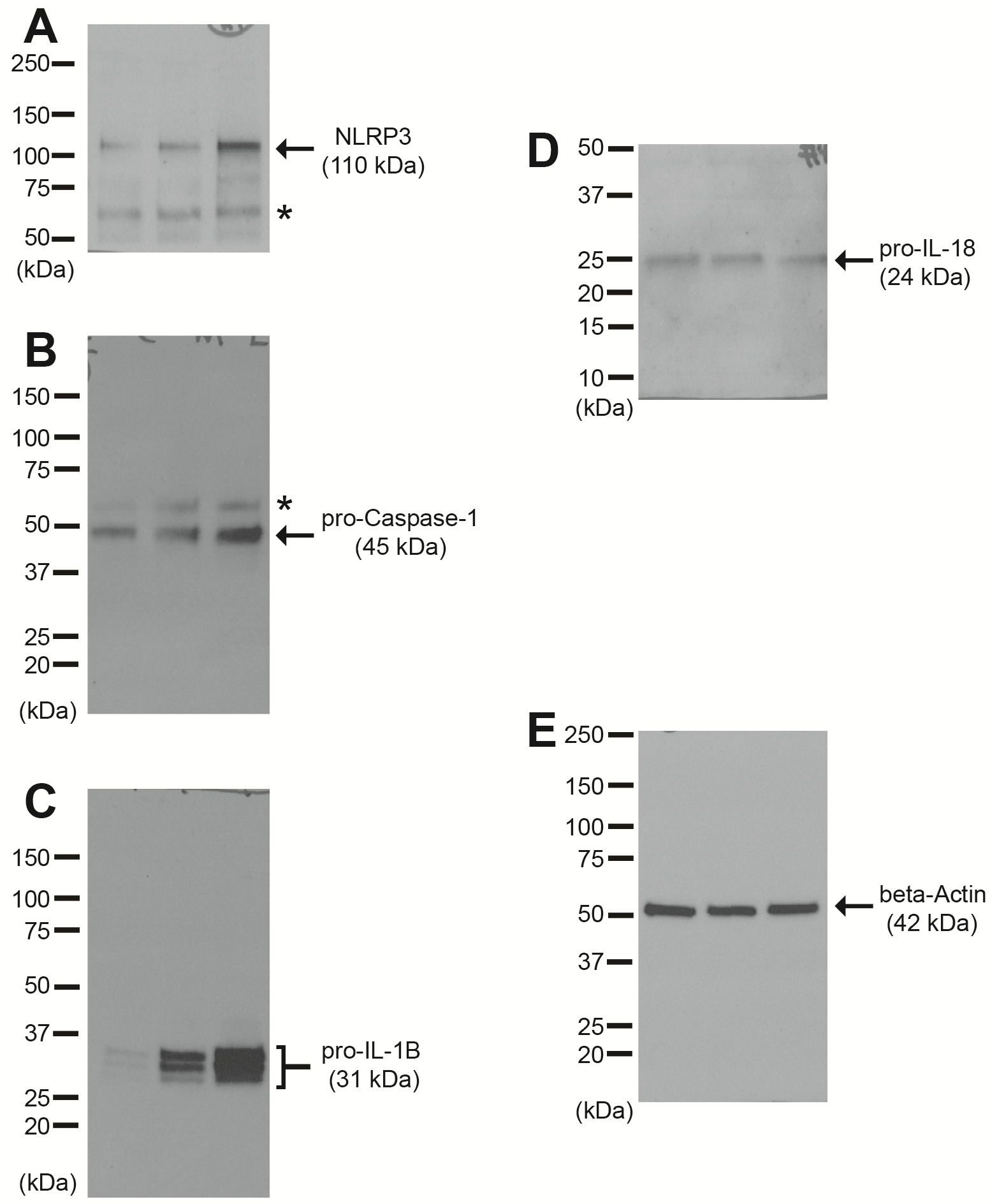


**Full-length gel images of Figure 6C**. Western blotting data using anti-NLRP3 (A), anti-Caspase-1 (B), anti-IL-1B (C), anti-IL-18 (D) and anti-beta-Actin antibodies. Molecular weight was indicated based on the dual color marker (Bio-Rad). * indicates non-specific band.
